# Electroacupuncture ameliorates neuroinflammation by inhibiting TRPV4 channel in ischemic stroke

**DOI:** 10.1111/cns.14618

**Published:** 2024-02-09

**Authors:** Xueqi Ren, Xinyi Gao, Ziqing Li, Yangyang Ding, Ao Xu, Lixia Du, Yufang Yang, Deheng Wang, Zhifei Wang, Shi Shu

**Affiliations:** ^1^ School of Traditional Chinese Medicine Shanghai University of Traditional Chinese Medicine Shanghai China; ^2^ School of Integrative Medicine Shanghai University of Traditional Chinese Medicine Shanghai China

**Keywords:** cerebral ischemia, electroacupuncture, microglia/macrophages, TRPV4

## Abstract

**Aims:**

We investigated the potential mechanisms underlying the therapeutic efficacy of electroacupuncture (EA) at the Shuigou (GV26) and Baihui (GV20) acupoints in the treatment of ischemic stroke.

**Methods:**

We assessed the therapeutic effects of EA on MCAO mice through behavioral studies and TTC staining. Various techniques, such as RT‐PCR, immunofluorescence, and Western blots, were employed to evaluate the activation and polarization of microglia/macrophages, and changes in the TRPV4 ion channel. We used the TRPV4 antagonist GSK2193874 (GSK219) to verify the involvement of TRPV4 in the therapeutic effects of EA.

**Results:**

EA effectively improved neurological impairments and reduced cerebral infarction volume in MCAO mice. It suppressed activated microglia/macrophages and inhibited their polarization toward the M1 phenotype post‐MCAO. EA also downregulated the expression of pro‐inflammatory cytokines, including *Tnf‐α*, *Il‐6*, *Il‐1β*, and *Ccl‐2* mRNA. Furthermore, EA reduced the elevated expression of TRPV4 following MCAO. Treatment with the TRPV4 antagonist GSK219 mirrored the effects of EA in MCAO mice. Notably, the combination of EA and GSK219 did not demonstrate an additive or synergistic effect.

**Conclusion:**

EA may inhibit neuroinflammation and exhibit a protective effect against ischemic brain injury by suppressing TRPV4 and the subsequent M1 polarization of microglia/macrophages.

## Graphical abstract

Electroacupuncture may inhibit neuroinflammation and exhibit a protective effect against ischemic brain injury by suppressing TRPV4 and the subsequent M1 polarization of microglia/macrophages. The Graphical Abstract was created with Figdraw (www.fgdraw.com).

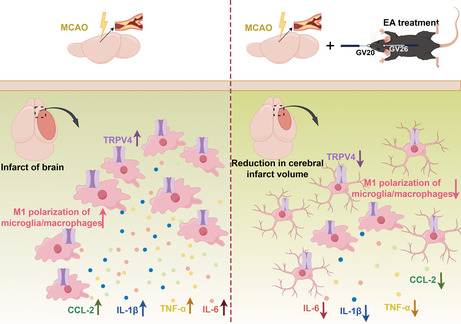

## 1 INTRODUCTION

Stroke is the second leading cause of death and disability globally, with ischemic stroke accounting for 80% of cases. The only FDA‐approved treatment, tissue plasminogen activator, must be given within 4.5 h of symptom onset, presenting challenges for timely treatment. Nowadays, there is still a lack of safe and effective therapies for ischemic stroke.

Neuroinflammation is an important component of the pathological process of ischemic stroke and is present in almost all stages of this disease.^1^ After occlusion, the affected brain area experiences glucose and oxygen deprivation, causing neuronal damage. This leads to the rapid activation of microglia cells, which accumulate at the site of injury in response to released neurotransmitters, free radicals, and other substances. The release of pro‐inflammatory mediators in this process serves as markers of neuroinflammation.^2^, ^3^ The activation of microglia cells can be detected in acute, subacute, and chronic stages of ischemic stroke and is correlated with the severity of ischemia.^4^ Activated microglia cells polarize into M1 and M2 phenotypes. M1 microglia cells produce pro‐inflammatory factors and cytotoxic substances, including tumor necrosis factor‐α (TNF‐α), interleukin‐6 (IL‐6), and IL‐1β, which contribute to neuroinflammation, blood–brain barrier leakage, and brain damage.^4^, ^5^ In contrast, M2 microglia cells secrete anti‐inflammatory factors and growth factors, such as IL‐10 and transforming growth factor‐β (TGF‐β), promoting angiogenesis and synaptic remodeling.^6^ Meanwhile, peripheral infiltrated macrophages enter into the brain through the blood–brain barrier and accumulate at the site of ischemia.^7^

Transient receptor potential vanilloid 4 (TRPV4) is an ion channel protein expressed in neurons and glial cells, involved in sensing and responding to temperature, pressure, chemicals, and mechanical stimuli.^8^, ^9^ Recent studies have linked TRPV4 to neuroinflammation. Activation of TRPV4 can induce microglia activation in mice, leading to the upregulation of pro‐inflammatory cytokines, including IL‐1β, TNF‐α, and IL‐6, intensifying the neuroinflammatory response.^10^, ^11^ Inhibiting TRPV4 may lead to remodeling of the cytoskeleton in microglia cells, thereby influencing their migration and morphology. This inhibition also reduces the secretion of IL‐1β and TNF‐α by glial cells, thereby alleviating the occurrence of neuroinflammation and ultimately reducing the likelihood of neuronal apoptosis.^12^, ^13^ Therefore, inhibiting TRPV4‐mediated neuroinflammation may be a promising therapeutic strategy for ischemic stroke treatment.

In traditional Chinese medicine theory, Baihui (GV20) is closely related to the brain and is commonly used to treat diseases of the head and face. Shuigou (GV26) is an emergency acupoint frequently used in stroke first aid. Electroacupuncture (EA) at the Shuigou (GV26) and Baihui (GV20) acupoints is widely used in clinical practice for the treatment of various conditions such as stroke, vascular dementia, and pediatric behavioral disorders. It has demonstrated positive therapeutic effects.^14^, ^15^, ^16^ Studies have shown that EA at Shuigou (GV26) and Baihui (GV20) can effectively increase TGF‐β1 levels in the serum, improve cerebral blood flow, and reduce the expression of glial maturation factor‐β. These effects contribute to the protection of EA against brain damage after cerebral ischemia–reperfusion injury.^17^, ^18^, ^19^ This study explores how EA at Shuigou (GV26) and Baihui (GV20) acupoints can treat ischemic stroke. We will investigate if EA can alleviate neuroinflammation in the ischemic brain of mice with cerebral ischemia and reperfusion injury. Additionally, we will examine the potential connection between anti‐neuroinflammatory effects and TRPV4 inhibition on microglia/infiltrated macrophages.

## 2 MATERIALS AND METHODS

### 2.1 MCAO surgery, drug, and EA treatment

All animal protocols and procedures were approved by the Experimental Animal Ethical Committee, Shanghai University of Traditional Chinese Medicine. Male C57BL/6J mice (22–26 g) were purchased from Vital River Laboratory Animal Technology Co., Ltd. (Beijing, China). Using isoflurane anesthesia, the mouse was prepared with a model of middle cerebral artery occlusion by inserting a filament through the right common carotid artery. After 30 min, the filament was removed. The mouse was kept warm at 37°C during the surgery and postoperative period until it regained consciousness.^20^ EA was administered for 20 min immediately after reperfusion at Shuigou (GV26) and Baihui (GV20) acupoints, followed by additional sessions at 24‐h intervals for up to 3 days. The EA parameters were as follows: density wave, 4/20 HZ; voltage range of 1–3 V, current range of 1–3 mA; with local slight jitter as the degree. GSK2193874 (GSK219, purity >98%, MCE, USA), a TRPV4 antagonist, was dissolved in saline containing 5% DMSO. GSK219 (5 mg/kg, i.p.) was administered once daily for three consecutive days prior to MCAO.

### 2.2 Brain infarct volume measurement

The brain was sliced into six coronal sections, each measuring 1 mm in thickness. These sections were soaked in 2% 2,3,5‐triphenyltetrazolium chloride (TTC, Sigma‐Aldrich, USA) at 37°C and then fixed with 4% paraformaldehyde. The infarct area in white was analyzed with ImageJ (free download at http://rsbweb.nih.gov/ij/).

### 2.3 Behavioral tests

As previously mentioned, on the first and third days after reperfusion, mice were subjected to the accelerated rotarod test and neurological deficit scoring after EA treatment to evaluate the effects of EA treatment on neurological functional deficits induced by MCAO.^20^

### 2.4 Quantitative real‐time PCR

RNA extraction and real‐time PCR were performed using the methods described earlier.^20^ The primer sequences for *Tnf‐α*, *Il‐1β*, *Il‐6*, and *β‐actin* were previously provided.^20^ The primer sequence of *Ccl2* was listed as GCTACAAGAGGATCACCAGCAG (forward), GTCTGGACCCATTCCTTCTTGG (reverse), *Cd206* as GTTCACCTGGAGTGATGGTTCTC (forward), GTTCACCTGGAGTGATGGTTCTC (reverse), *Arg‐1* as CATTGGCTTGCGAGACGTAGAC (forward), GCTGAAGGTCTCTTCCATCACC (reverse), *Il‐10* as GCCCTTTGCTATGGTGTC (forward), TCTCCCTGGTTTCTCTTCC (reverse), *Tgf‐ β* as TGGGGACTTCTTGGCA (forward), ATAGGGGCGTCTGAGGAACCT (reverse), *Trpv4* as CCTGCTGGTCACCTACATCA (forward), CCTGCTGGTCACCTACATCA (reverse).

### 2.5 Immunofluorescence staining

Brain slices were prepared as previously described,^21^ and 0.3% Triton ×100 was used to penetrate 15 min. Brain slices (30 μm) were blocked with QuickBlock™ (Beyotime, China) for 1 h, and then incubated in a refrigerator with primary antibodies at 4°C overnight. We performed single immunofluorescence staining using rabbit anti‐Iba1 (1:1000, Abcam, USA). Performed double immunofluorescence staining using mouse anti‐Iba1 (1:100, Santa Cruz Biotechnology, USA) with rabbit anti‐TRPV4 (1:200, Abcam, USA), rabbit anti‐Iba1 (1:1000, Abcam, USA) with mouse anti‐CD86 (1:50, Santa Cruz Biotechnology, USA), and rabbit anti‐Iba1 (1:1000, Abcam, USA) with goat anti‐CD206 (1:250, R&D Systems, USA). After washing the brain slices with PBS containing 0.3% Triton ×100, Alexa 594‐conjugated goat anti‐rabbit antibodies (1:1000, Life Technologies Corporation, USA), Alexa 555‐conjugated donkey anti‐goat antibodies (1:1000, Life Technologies Corporation, USA), and Alexa 488‐conjugated goat anti‐rabbit antibodies (1:1000, Life Technologies Corporation, USA) were added and incubated at room temperature for 2 h. The nucleus was stained with DAPI. Using laser scanning confocal microscopy (Leica SP‐8) to take pictures. ImageJ was used for analysis.

### 2.6 Western blotting

The protein levels of iNOS and TRPV4 were detected by Western blotting.^20^ The primary antibodies were as follows: rabbit anti‐iNOS (1:1000, Cell Signaling Technology, USA), rabbit anti‐TRPV4 (1:500, Abcam, USA), and mouse anti‐β‐actin (1:5000, Sigma‐Alrich).

### 2.7 Sholl analysis

Sholl analysis was performed using the Sholl analysis plugin that comes with ImageJ. During the analysis, the microglia/macrophages body was taken as the center of the circle, and a series of concentric circles were drawn to obtain the number of protrusion intersections that changed with the distance from the central region, so as to reflect the complexity of the cell.

### 2.8 Statistical analysis

Data are expressed as mean ± SEM. Statistical analysis was performed using GraphPad Prism 9. We conducted a Kolmogorov–Smirnov test to assess the normality of the data. When the data met the assumptions of normality and homogeneity of variance, we employed Student's *t*‐test, ANOVA followed by Tukey's post hoc test or two‐way repeated measured ANOVA with a post hoc Bonferroni test. For data that did not follow a normal distribution, we used the Mann–Whitney *U* or Kruskal‐Wallis test followed by Dunn's post hoc test. For the data that conformed to normal distribution but did not conform to homogeneity of variance, we used Brown‐Forsythe and Welch ANOVA test. For multiple comparisons, we also used either the *t*‐test or Mann–Whitney *U* test, followed by Bonferroni correction. *p* < 0.05 was considered statistically significant.

## 3 RESULTS

### 3.1 EA ameliorated neurological functional deficits and reduced cerebral infarction in MCAO mice

**FIGURE 1:**
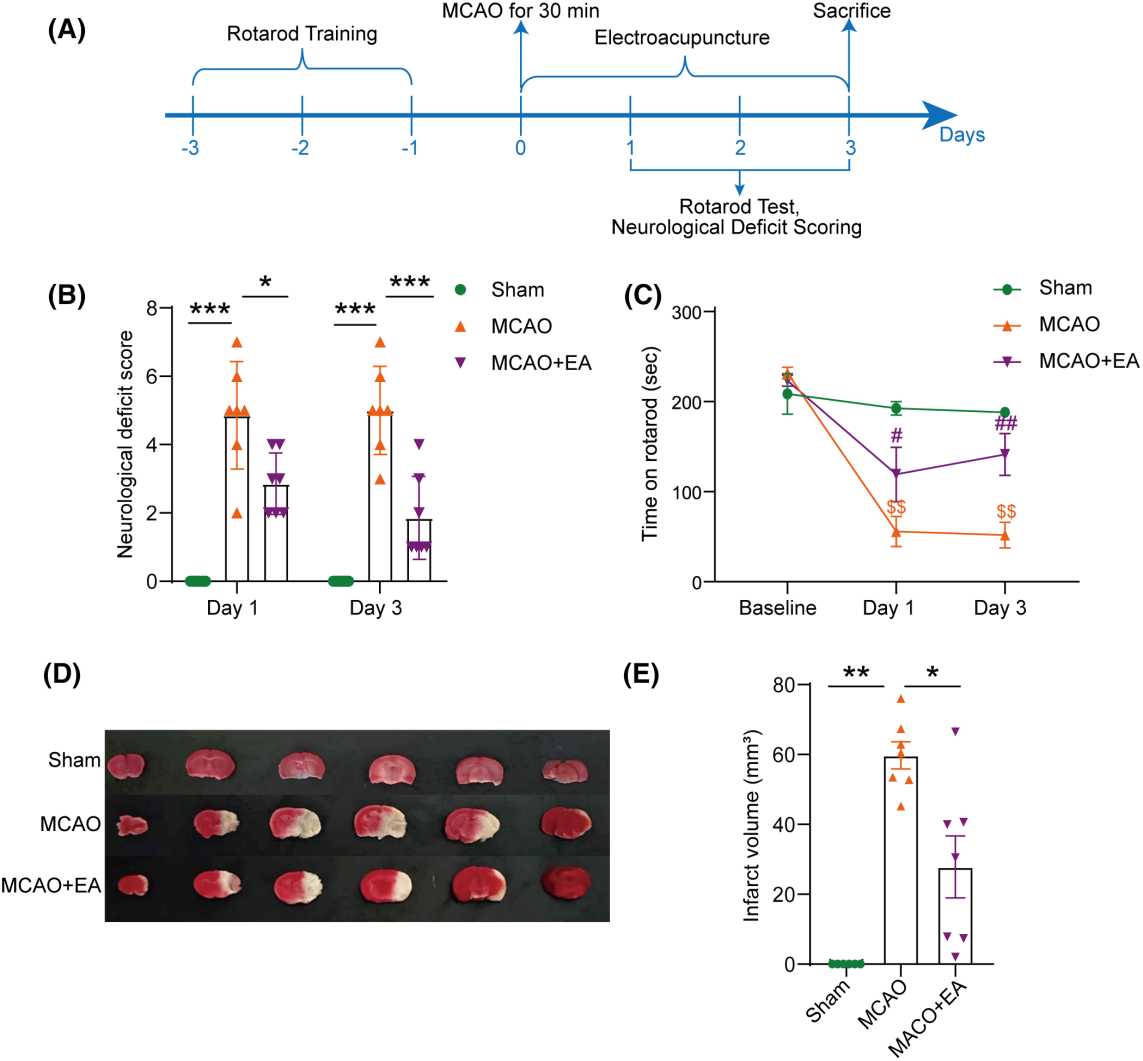
EA ameliorated neurological functional deficits and reduced cerebral infarction in MCAO mice. (A) Timeline for EA treatment and behavioral testing. (B) Compared with the MCAO group, there was a significant decrease in the neurological deficit scores on the first and third days after EA treatment. (C) EA significantly increased the retention time of MCAO mice on the rotating rod on the first and third days after reperfusion. (D, E) EA significantly reduced the volume of cerebral infarction in MCAO mice on the third day. *p < 0.05, **p < 0.01, ***p < 0.001; for rotarod test, ^$$^ *p* < 0.01 compared with the sham control group; ^#^ *p* < 0.05, ^##^ *p* < 0.01 compared with the MCAO group; *n* = 6–7 per group.

The experiments were conducted as depicted in Figure 1A. On the first and third days after reperfusion, we assessed the protective effect of acupuncture treatment on MCAO mice. MCAO significantly reduced the time that mice spent on an accelerating rotarod, while EA significantly increased the retention time on the first day and the third day (Figure 1B). At the same time, the neurological deficit score of mice significantly increased after MCAO, but EA effectively reduced the score on the first day and the third day (Figure 1C). Compared with the MCAO group, the brain infarct volume in the EA‐treated group significantly decreased (Figure 1D,E).

### 3.2 EA inhibited the activated microglia/macrophages in the ischemic cerebral cortex

**FIGURE 2:**
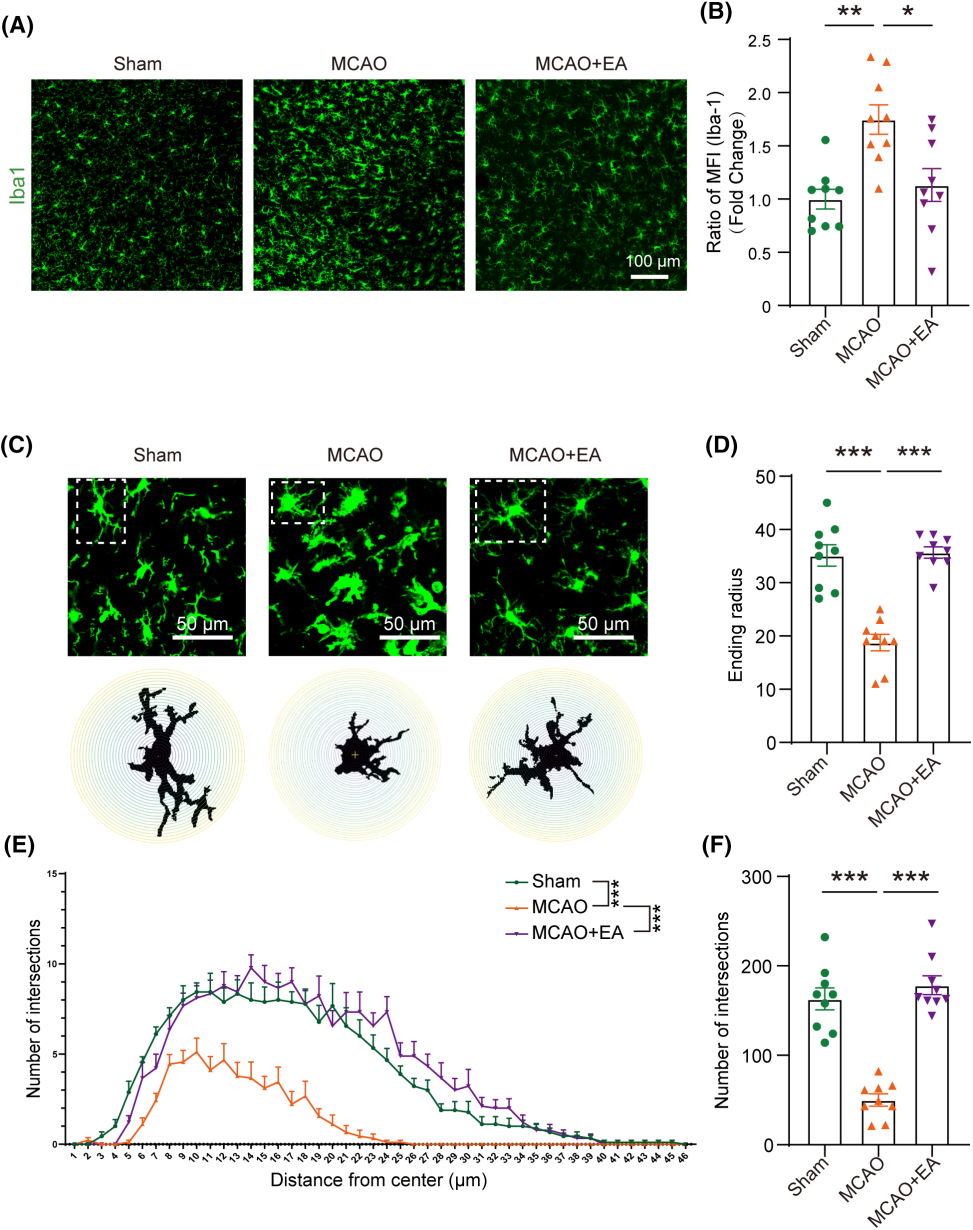
EA inhibited the activated microglia/macrophages in ischemic cerebral cortex. Immunofluorescence results showed increased fluorescence intensity in the MCAO group, which was effectively reversed by EA treatment (A and B). Microglia/macrophages morphology was analyzed using Sholl analysis (C). Compared to the MCAO group, the EA group exhibited an increase in the farthest reached radius (D), microglia/macrophages intersections per radius (E), and total intersections (F). *p < 0.01; ***p < 0.001; *n* = 9 from 3 mice per group.

Immunofluorescence analysis showed that, compared with the MCAO group, the number of Iba1^+^ cells was significantly reduced after EA (Figure 2A). Subsequently, we conducted a statistical analysis of fluorescence intensity, which further confirmed that EA could inhibit the activated microglia/macrophages (Figure 2B). The morphology of microglia/macrophages was analyzed by Sholl analysis using ImageJ, and the distance between each concentric circle is 1 μm. The results showed that after MCAO, microglia/macrophages exhibited an activated state characterized by shortened processes, reduced branching, and enlarged cell bodies. EA effectively inhibited the activation of microglia/macrophages (Figure 2C). The maximum radius reached by microglia/infiltrated macrophages processes in the ischemic brain cortex of mice in the MCAO group significantly decreased. However, EA treatment reversed this phenomenon (Figure 2D). Compared with the MCAO group, the number of microglia/ macrophages intersections per radius increased after EA treatment (Figure 2E). The number of intersections between microglia/macrophages processes and equidistant concentric circles significantly increased after EA treatment (Figure 2F). These results suggested that EA could inhibit the activated microglia/macrophages in the ischemic cerebral cortex.

### 3.3 EA inhibited the polarization of M1 microglia/macrophages in the ischemic cerebral cortex

**FIGURE 3:**
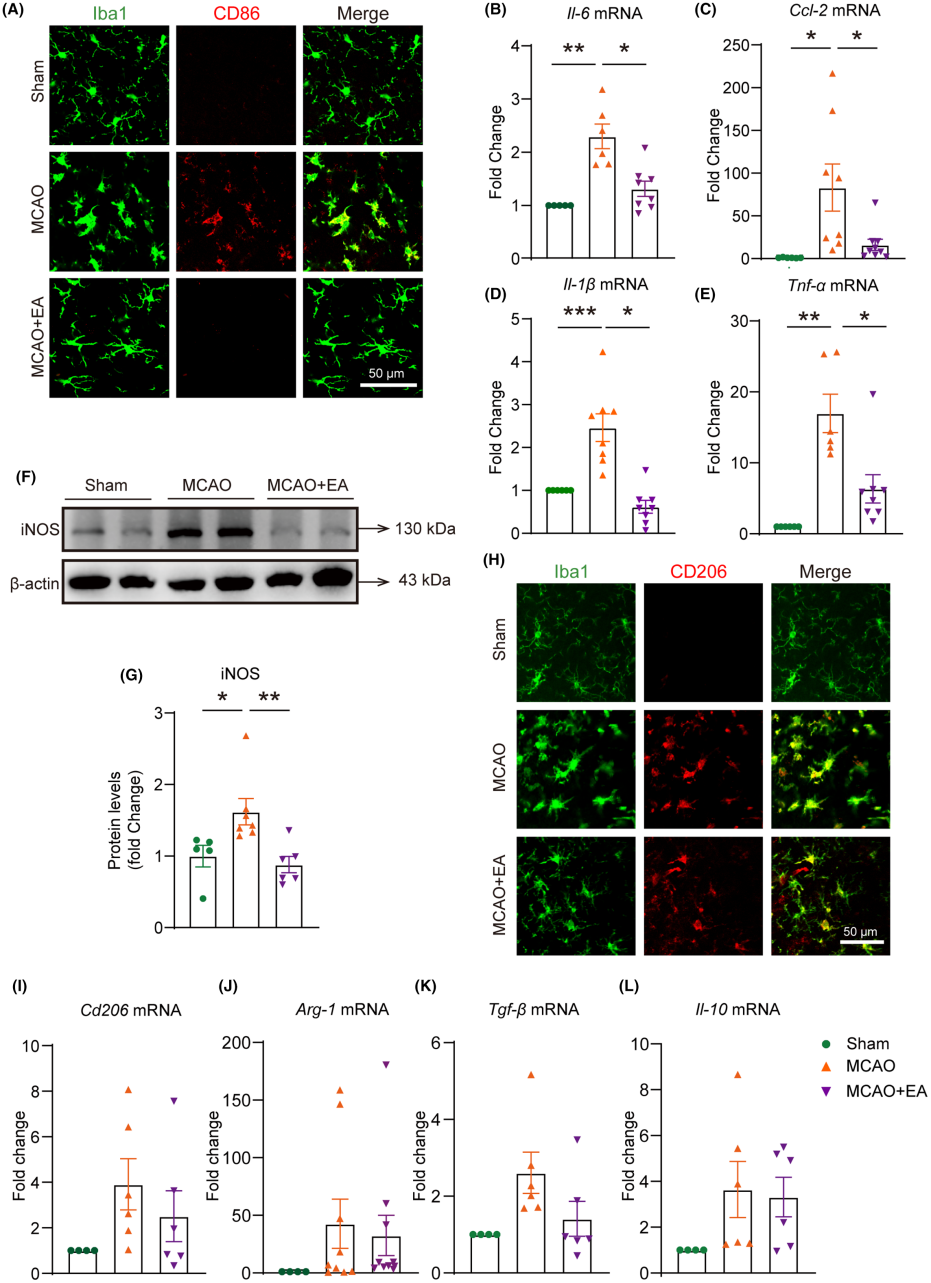
EA inhibited the polarization of M1 microglia/macrophages in the ischemic cerebral cortex. (A) Immunofluorescence showed that EA reduced the number of Iba1^+^ CD86^+^ cells in the ischemic cerebral cortex. (B–E) After MCAO, the mRNA expression of pro‐inflammatory cytokines *Ccl‐2*, *Il‐6*, *Tnf‐α*, and *Il‐1β* increased. EA treatment suppressed this upregulation. (F, G) EA treatment inhibited the elevated protein levels of iNOS after MCAO. (H) Immunofluorescent staining revealed iba1^+^ and CD206^+^ cells in the ischemic cerebral cortex of the Sham group, MCAO group, and EA group. (I–L) Compared with the MCAO group, after EA treatment, there was no statistically significant difference in the mRNA expression levels of *Tgf‐β*, *Il‐10*, *Cd206*, and *Arg‐1* secreted by M2 microglia/macrophages. *p < 0.05; **p < 0.01; ***p < 0.001; *n* = 4–8 per group.

Next, we performed co‐labeling of Iba1 (a marker of microglia/macrophages) with CD86 (a marker of M1 microglia/macrophages). The results revealed a noteworthy increase in the number of Iba1^+^ and CD86^+^ cells in the MCAO group, and this effect was significantly reduced by EA (Figure 3A). We also detected the mRNA levels of M1‐secreted cytokines such as *Ccl‐2*, *Il‐6*, *Tnf‐α*, and *Il‐1β*. The results demonstrated that, compared with sham controls, the mRNA levels of *Ccl‐2*, *Il‐6*, *Tnf‐α*, and *Il‐1β* in the ischemic cerebral cortex increased significantly after MCAO. As expected, treatment with EA significantly down‐regulated the mRNA expression of these cytokines respectively (Figure 3B–E). The level of iNOS protein in the cerebral cortex of mice was also significantly increased 3 days after MCAO, and this upregulation was markedly down‐regulated by EA treatment (Figure 3F,G). Similarly, we initially performed co‐labeling of CD206 (M2 microglia/macrophages marker) with Iba1. The results showed a decreasing trend in the number of CD206^+^ and Iba1^+^ cells in the EA group compared to the MCAO group (Figure 3H). Subsequently, we assessed the mRNA levels of cytokines *Tgf‐ β, Il‐10, Cd206*, and *Arg‐1* secreted by M2 microglia/macrophages. The results demonstrated that following MCAO operation, there was an upregulation in mRNA levels of *Tgf‐β*, *Il‐10*, *Cd206*, and *Arg‐1*. There was no statistically significant difference between the EA group and the MCAO group in these mRNA levels (Figure 3I–L). Therefore, our findings suggested that EA only inhibited the polarization of M1 microglia/macrophages in the ischemic cerebral cortex.

### 3.4 EA inhibited the upregulated TRPV4 in the ischemic cerebral cortex

**FIGURE 4:**
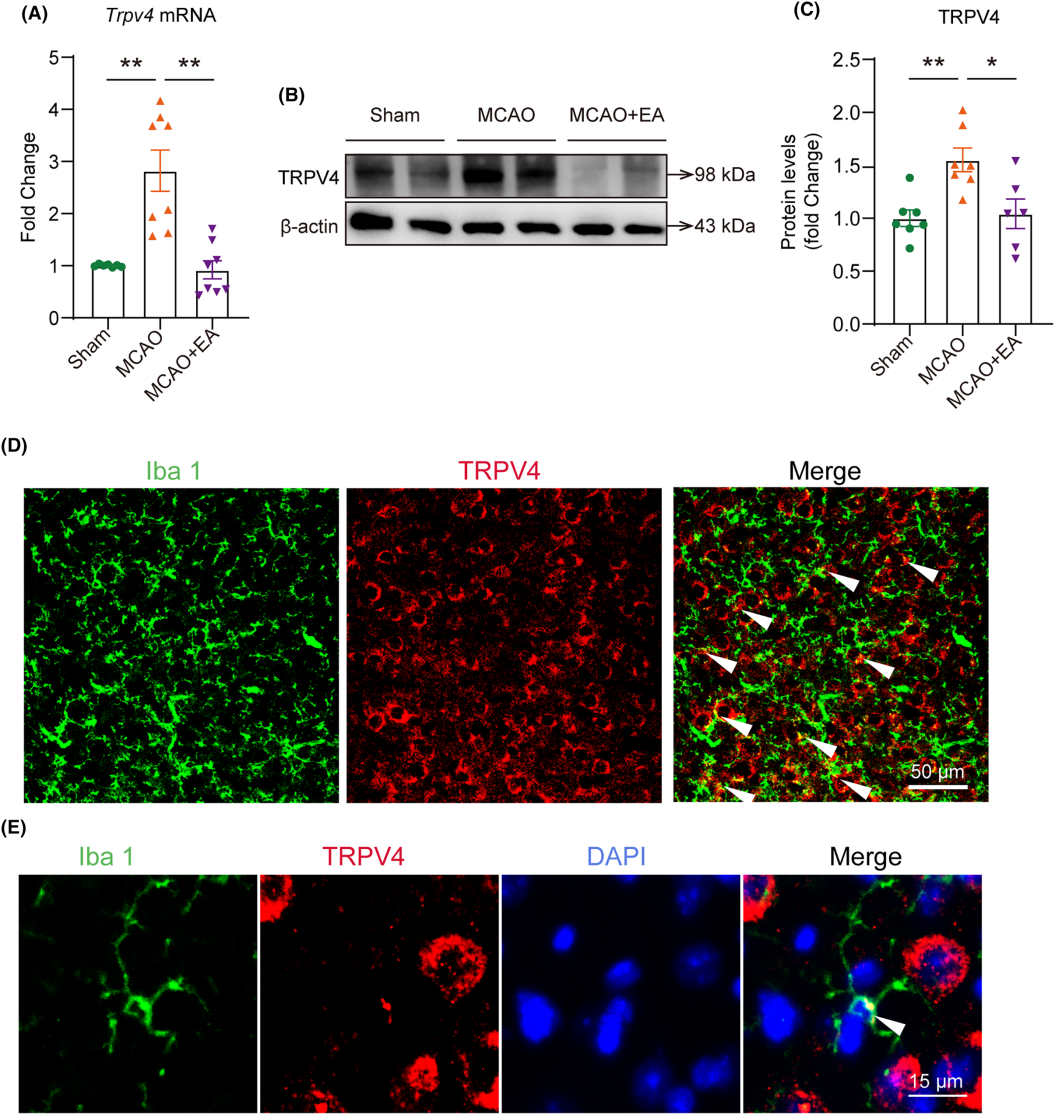
EA inhibited the upregulated TRPV4 in the ischemic cerebral cortex. (A–C) After MCAO, both the protein and mRNA expression levels of TRPV4 significantly increased. This upregulation was completely suppressed by EA treatment. (D) A representative image of immunofluorescence co‐labeling of Iba1 with TRPV4. (E) A representative image of an Iba1^+^ TRPV4^+^ cell in the normal mouse cerebral cortex. The white arrow indicates where the co‐labeling is. *p < 0.05; **p < 0.01; ***p < 0.001; *n* = 6–8 per group.

We examined the effects of EA on mRNA and protein expression levels of TRPV4 in the ischemic cerebral cortex. The results showed that there was a significant increase in TRPV4 mRNA and protein expression in the infarcted cortex of mice. EA robustly suppressed this upregulation of TRPV4. After EA treatment, the mRNA and the protein expression of *Trpv4* decreased (Figure 4A–C). To confirm the expression of TRPV4 on microglia/macrophages, we performed immunofluorescence co‐staining of TRPV4 with Iba1. We observed Iba1^+^ TRPV4^+^ cells in the normal mouse cerebral cortex, indicating that TRPV4 was expressed on microglia/macrophages (Figure 4D,E).

### 3.5 Inhibition of TRPV4 may contribute to the effects of EA on ameliorating the neurological functional deficits in MCAO mice

**FIGURE 5:**
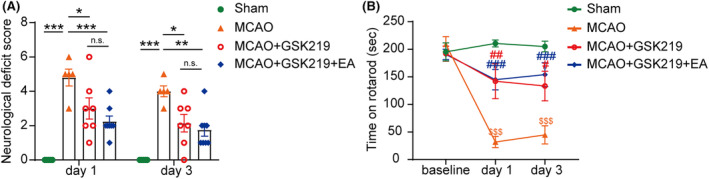
Inhibition of TRPV4 may contribute to the effects of EA on ameliorating the neurological functional deficits in MCAO mice. (A) On the first and third day after MCAO, both GSK219 and GSK219 + EA reduced the neurological deficit scores in MCAO mice, and there was no statistically significant difference between the two groups. (B) On the first and third days after MCAO, GSK219 and GSK219 + EA prolonged the time that MCAO mice spent on the accelerating rotarod, and there was no statistically significant difference between the two groups. *p < 0.05; **p < 0.01; ***p < 0.001; n.s., non‐statistical significance; *n* = 5–7 per group.

To confirm the involvement of TRPV4 in the therapeutic effects of EA on MCAO mice, we further conducted validation experiments using the TRPV4 antagonist GSK219 on the mice. We found that 5 mg/kg GSK219 robustly reduced MCAO‐upregulated *Trpv4* mRNA in the ischemic brain (Figure S1). On the first and third day after MCAO, the mice in the MCAO group exhibited a significant increase in neurological deficit scores compared with the sham group. This increase was reversed by treatment with the TRPV4 antagonist GSK219 as well as GSK219 + EA (Figure 5A). Similarly, on the first and third day after MCAO, the mice in the MCAO group exhibited a significant decrease in the time spent on the rotarod compared to the sham surgery group. As expected, treatment with GSK219, as well as GSK219 + EA, resulted in an extension of the time spent on the rotarod (Figure 5B). It is of interest to note that there was no statistical significance between the GSK219 group and GSK219 + EA group in terms of their effects on ameliorating neurological functional deficits in MCAO mice.

### 3.6 Suppressing the upregulation of TRPV4 may contribute to the anti‐neuroinflammatory effects of EA in the ischemic cerebral cortex

**FIGURE 6:**
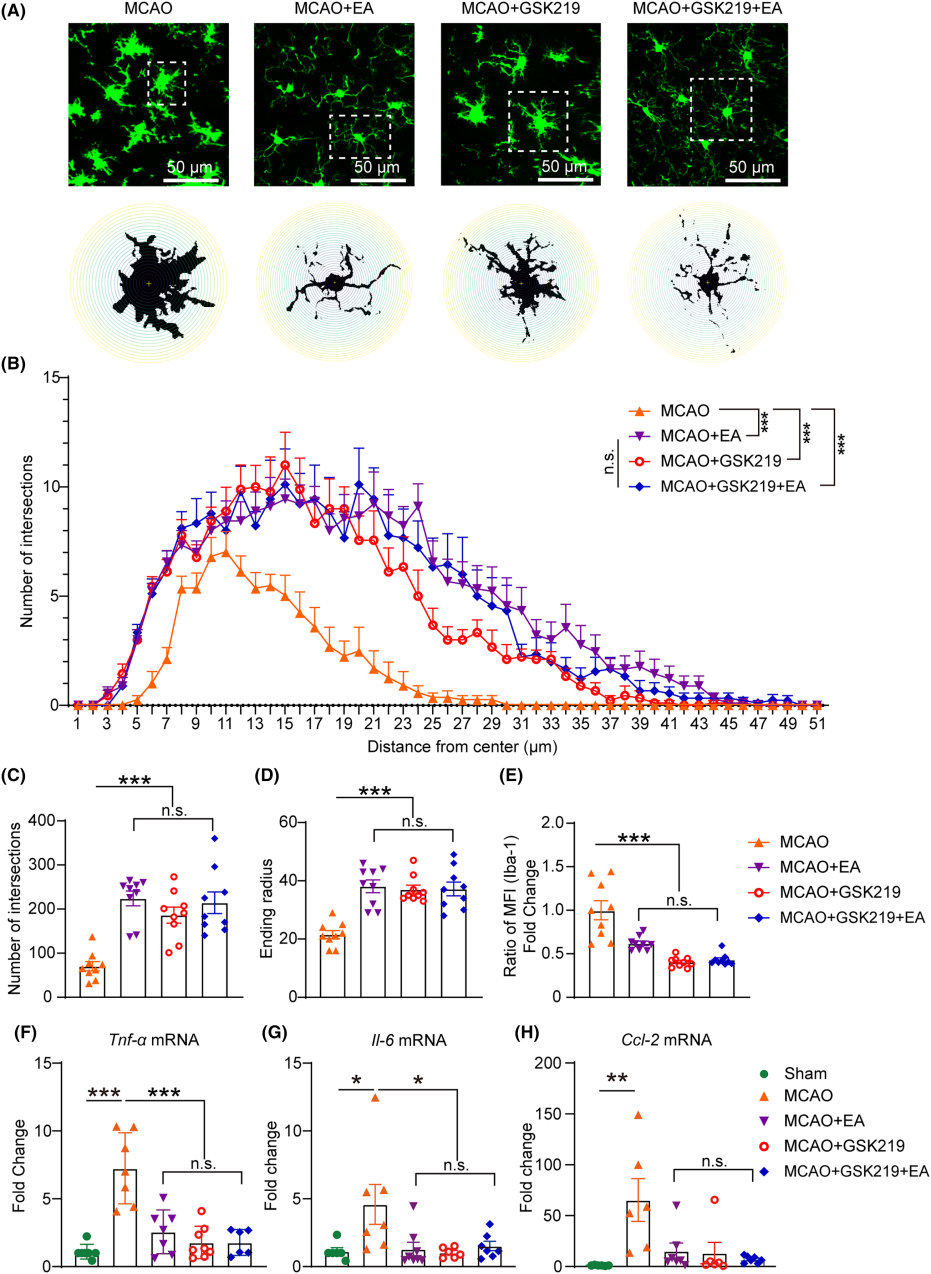
Suppressing the upregulation of TRPV4 may contribute to the anti‐neuroinflammatory effects of EA in the ischemic cerebral cortex. Sholl analysis quantified microglia/macrophages branching complexity. In the penumbra of the cerebral cortex, mice treated with GSK219, EA, and EA + GSK219 showed increased intersections per radius (A and B), total intersections (C), and farthest‐reaching circles (D). The fluorescence intensity significantly decreased in these three groups (E). The EA, GSK219, and GSK219 + EA groups exhibited similar therapeutic effects, inhibiting mRNA expression of *Tnf‐α*, *Il‐6*, and *Ccl‐2* (F–H). Statistical analysis revealed no significant differences among the EA, GSK219, and GSK219 + EA groups. *n* = 9 from 3 mice per group for Immunofluorescence. *p < 0.05; **p < 0.01; ***p < 0.001; n.s., non‐statistical significance; each group *n* = 6–8 for rt‐PCR.

The morphology of microglia/macrophages in the EA, GSK219, and GSK219 + EA groups was close to resting state (Figure 6A). Compared with that in the MCAO group, the number of intersections per radius (Figure 6B), the farthest radius reached by microglia/macrophages processes (Figure 6C), and the total number of intersections between microglia/macrophages and equidistant concentric circles (Figure 6D) all significantly increased in the EA, GSK219, and GSK219 + EA groups. In the statistical analysis of fluorescence intensity, compared to the MCAO group, the EA group, GSK219 group and the GSK219 + EA group exhibited a decrease (Figure 6E). Importantly, in terms of the number of intersections per radius, there was no statistical difference between the EA group and the GSK219 + EA group. Moreover, in terms of the farthest radius reached, the total number of intersections, and the statistical analysis of fluorescence intensity, there was no statistical difference among the EA, GSK219, and GSK219 + EA groups.

Subsequently, we further examined the mRNA expression levels of *Tnf‐α*, *Il‐6*, and *Ccl‐2*. The results showed that on the third day after MCAO, compared to the sham group, the MCAO group exhibited a significant upregulation of *Tnf‐α*, *Il‐6* and *Ccl‐2* in the ischemic cortex. However, the increase of *Tnf‐α* and *Il‐6* was robustly suppressed by EA, GSK219, and GSK219 + EA treatments, respectively (Figure 6F–H). These treatments also had the trend to reduce *Ccl‐2* mRNA in the ischemic brain. Similar to the Sholl analysis, no statistically significant differences were observed in the mRNA expression levels of *Tnf‐α*, *Il‐6*, and *Ccl‐2* among EA, GSK219, and GSK219 + EA groups. These findings indicated that GSK219 did not enhance the anti‐neuroinflammatory effects of EA in the ischemic cerebral cortex.

## 4 DISCUSSION

EA is commonly used in stroke rehabilitation, involving stimulating acupuncture points with electrical currents. This can improve symptoms and promote recovery. Electrical stimulation at these points has various effects such as pain relief, anti‐inflammatory properties, and enhanced blood circulation, aiding overall stroke rehabilitation.^22^ The combined utilization of Shuigou (GV26) and Baihui (GV20) acupoints is a well‐established approach in stroke treatment, exhibiting significant efficacy as supported by clinical evidence.^23^, ^24^ In our study, we observed that EA at Shuigou (GV26) and Baihui (GV20) points exhibited benefits in MCAO mice, leading to improvements in cerebral infarction volume and behavioral tests. We aimed to elucidate the underlying mechanism of this effect.

After ischemic stroke, microglia are activated and infiltrated macrophages migrate toward the site of injury, both polarizing into two distinct phenotypes, M1 and M2. In the pathological process of ischemic stroke, both M1 and M2 microglia/macrophages co‐exist and exert mutual antagonism. In this context, if the activation of M1 microglia/macrophages are enhanced, it can promote the occurrence of neuroinflammation. Conversely, enhancing the activation of M2 microglia/ macrophages can improve stroke outcomes. The delicate interplay between these two phenotypes determines the fate of injured neurons.^25^, ^26^ This study demonstrated that EA significantly inhibited the activated microglia/macrophages and the polarization to the pro‐inflammatory phenotype (M1) in the ischemic cerebral cortex, leading to down‐regulation of inflammatory factors such as IL‐6, TNF‐α, IL‐1β, and CCL‐2. However, our results also indicated that EA did not promote microglia/macrophages polarization toward an anti‐inflammatory phenotype (M2). This phenomenon may be attributed to the increased differentiation of M1 microglia/macrophages and the relatively low number of M2 microglia/macrophages in the early rapid phase of brain injury, 3 days after ischemic stroke. Our findings suggested that EA inhibited the polarization of microglia/macrophages toward the M1 phenotype, promoting the restoration of dynamic balance between M1 and M2 microglia/macrophages. This balance was instrumental in alleviating the inflammatory response and facilitating the repair of brain injury. Henceforth, this explains why EA had more pronounced effects on modulating the activity of M1 microglia/macrophages compared to its impact on regulating M2 microglia/macrophages.

TRPV4 is ubiquitously expressed in various tissues and immune cells and its activation leads to Ca^2+^ influx, cytoskeleton rearrangement, intracellular signal transduction, and gene expression changes influencing cell phenotype and function.^27^, ^28^ In the central nervous system, TRPV4 has been identified in neurons, astrocytes, and microglia.^8^ In our study, we confirmed that TRPV4 was expressed in microglia/macrophages. Studies have revealed the use of TRPV4 antagonists can significantly reduce the occurrence of neuroinflammation in diseases such as Parkinson's disease or depression.^29^, ^30^ An upregulation of TRPV4 expression in the ipsilateral brain following ischemic injury results in increased neuronal damage.^31^, ^32^ In various pain models, EA can suppress the upregulation of TRPV4 and TRPV1 expression in the spinal cord or brain, thereby exerting therapeutic effects.^33^, ^34^, ^35^ Our study observed a significant increase in both TRPV4 mRNA expression and protein levels following MCAO modeling; however, EA treatment effectively reversed this process.

Microglia, as the resident infiltrated macrophages of the central nervous system, have been extensively studied in relation to TRPV4. According to the literature, the expression levels of cytokines such as IL‐1β, TNF‐α, and IL‐6 contribute to neuroinflammation‐mediated neuronal death, while inhibition of TRPV4 expression subsequently leads to the suppression of NF‐κB activation, reduces secretion of glial cell‐derived IL‐1β and TNF‐α, and mitigates neuronal apoptosis.^13^ Our research indicated that treatment with the TRPV4 antagonist GSK219 improved functional deficits in MCAO mice and downregulated pro‐inflammatory factors secreted by M1 microglia/macrophages similar to the effects observed at EA Shuigou (GV26) and Baihui (GV20) acupoints. Furthermore, the combined administration of GSK219 and EA did not exhibit an additive or synergistic effect, suggesting that EA may exert its therapeutic role by inhibiting the upregulation of TRPV4 expression after MCAO.

It is of note that TRVP4 is widely expressed in neurons and glial cells in the brain. Activation of TRPV4 significantly increases neuronal excitability,^36^ and enhances neuronal excitability which leads to an increase in extracellular ATP, resulting in targeted motility of microglia.^37^ Upregulation of TRPV4 also activates astrocytes,^29^ and activated astrocytes can promote microglial activation and modulate their functions. Conversely, the activated microglia can also stimulate the activation of astrocytes.^38^, ^39^ Therefore, TRPV4 derived from neurons or astrocytes may also play a role in EA's effect on polarization of microglia/macrophages in the ischemic brain. This notion needs to be further investigated.

Overall, our study highlights the efficacy of EA at Shuigou (GV26) and Baihui (GV20) on MCAO mice while proposing a mechanism involving inhibition of the TRPV4 channel to suppress M1 polarization in microglia/macrophages following ischemic stroke in mice. These findings provide a potential mechanism for EA's therapeutic effects on ischemic stroke treatment at Shuigou (GV26) and Baihui (GV20), offering valuable theoretical support for clinical applications.

## AUTHOR CONTRIBUTIONS

SS and ZW designed the experiments. XR, XG and ZL performed the experiments. XR, YD, AX, LD, YY and DW analyzed and interpreted the final data. XR wrote the manuscript. SS and ZW revised the manuscript. All authors have read and approved the final manuscript.

## FUNDING INFORMATION

This study was supported by grants from the National Natural Science Foundation of China (82374581 & 81774388 to SS, 81873029 to WZ, 82104640 to YY).

## CONFLICT OF INTEREST STATEMENT

The authors declare that the research was conducted in the absence of any commercial or financial relationships that could be construed as a potential conflict of interest.

## Supporting information


Figure S1.

## Data Availability

The data that support the findings of this study are available from the corresponding author upon reasonable request.
