# Supplementary material for: Electroacupuncture ameliorates neuroinflammation by inhibiting TRPV4 channel in ischemic stroke
Source: CNS Neurosci Ther. 2024 Feb 9;30(2):e14618. doi: 10.1111/cns.14618 (PMC10853892; doi:10.1111/cns.14618)
Supplement: Supplementary file 1 — Figure S1. [file CNS-30-e14618-s001.pdf]

Manuscript ID: CNSNT-2023-1438

Electroacupuncture ameliorates neuroinflammation by inhibiting TRPV4 channel in ischemic stroke

Xueqi Ren[1], Xinyi Gao[2], Ziqing Li[2], Yangyang Ding[2], Ao Xu[2], Lixia Du[2], Yufang Yang[2], Deheng Wang[2], Zhifei Wang[2]*, Shi Shu[1]*

Full unedited blot for Figure 3H

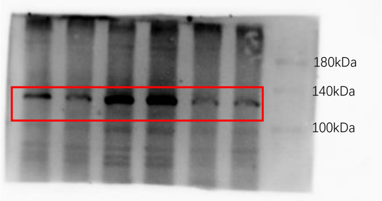

iNOS (130 kDa)

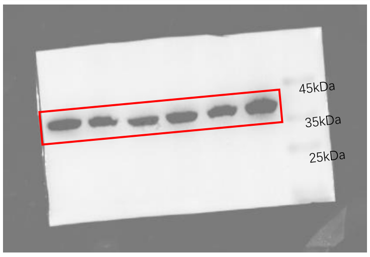

β-actin (43 kDa)

Full unedited blot for Figure 4B

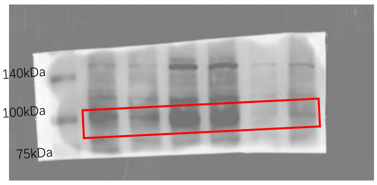

TRPV4 (98 kDa)

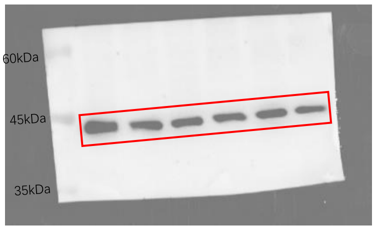

β-actin (43 kDa)

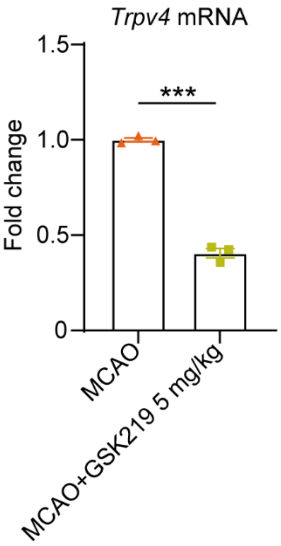

Figure S1 GSK219 inhibited the upregulation of *Trpv4* mRNA expression in the brain after MCAO. 5 mg/kg GSK219 robustly reduced MCAO-upregulated *Trpv4* mRNA in the ischemic brain. *** $p < 0.001$.
